# Molecular Origin of Interfacial Anomalies in Azeotropic Refrigerant Mixtures

**DOI:** 10.1021/acs.jpcc.5c04188

**Published:** 2025-08-04

**Authors:** Ismail I. I. Alkhatib, Carlos G. Albà, Yuting Li, Simon Stephan, Fèlix Llovell, Lourdes F. Vega

**Affiliations:** † Research and Innovation Center on CO_2_ and Hydrogen (RICH Center) and Chemical and Petroleum Engineering Department, 105955 Khalifa University of Science and Technology, PO Box 127788, Abu Dhabi, United Arab Emirates; ‡ Department of Chemical Engineering, ETSEQ, 16777 Universitat Rovira i Virgili (URV), Campus Sescelades, Av. Països Catalans 26, 43007 Tarragona, Spain; § GasN2, Avda. Diagonal 579, 08014 Barcelona, Spain; ∥ Molecular Thermodynamics Group (MTG), RPTU Kaiserslautern, Erwin-Schrodinger-Strasse 44, 67663 Kaiserslautern, Germany

## Abstract

Interfacial anomalies such as the formation of an aneotrope have been well-established for binary refrigerant blends with polar and nonpolar compounds. This remains largely unexplored for mixtures of polar refrigerants. This study investigates the phase equilibria and interfacial properties of pure refrigerants and their binary mixtures, specifically focusing on the molecular origin of interfacial anomalies. The polar soft-SAFT equation coupled with density gradient theory (DGT) was used to model the interfacial properties of 16 pure refrigerants, as well as phase equilibria and surface tension of selected binary mixtures with available data. Once the model was validated, the phase equilibria and interfacial tension for mixtures with R134a were systematically predicted with emphasis on azeotrope and aneotrope formation. It was determined that, unlike polar + nonpolar refrigerant mixtures, the occurrence of azeotropy is not a strict prerequisite for manifesting aneotropic-like behavior. The aneotrope composition was always consistent with the composition at which zero relative adsorption was observed rather than the composition of the azeotrope. Distinct interfacial enrichment in these mixtures was absent, indicating a subtle preferential adsorption rather than significant accumulation at the interface. This research provides a deeper understanding of interfacial anomalies and molecular-level insights for the rational design of sustainable next-generation refrigerants.

## Graphical abstract

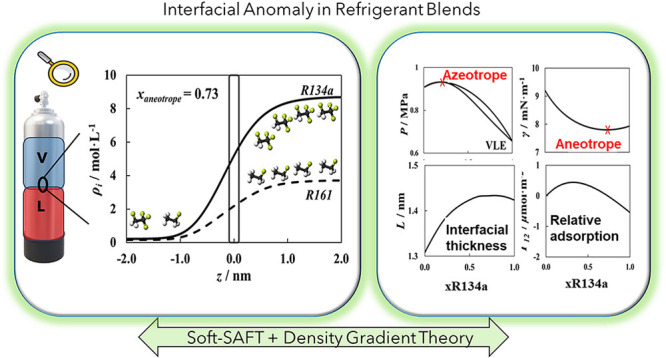

## Introduction

Refrigeration and air conditioning for objects and space cooling is one of the transformative technologies of the 20th century,^1^ becoming a necessity for human comfort in the face of the persistent increase in global surface temperatures—an outcome of global warming.^2^ A working fluid or refrigerant is the backbone of the refrigeration system, acting as a medium for transporting heat in the system with cyclical phase changes (e.g., evaporation with heat absorption and condensation with heat rejection). The choice of refrigerant went through several iterations over the past decades, shaped by their contribution to climate change. This originated from the use of chlorofluorocarbons (CFCs) with high ozone depletion potential (ODP) and propagated with hydrofluorocarbons (HFCs) which have global warming potential (GWP) significantly higher than CO_2_.^3–6^ Since the enforcement of Kigali’s amendment to the Montreal Protocol in 2019,^7^ several other regulations are already in effect to phase-out the utilization of currently used HFCs,^8–12^ which if adopted on a global-scale can potentially avoid cumulative 53 GtCO_2_-eq emissions by 2060.^8^

An ideal refrigerant capable of meeting required regulations and replacing HFCs should be nontoxic and nonflammable (or mildly flammable), have a null ODP combined with a low GWP (in line with regulations), and possess desirable thermophysical properties suitable for the intended application and operating conditions (e.g., low heat of vaporization, low boiling point, high critical temperature, etc.) necessary to obtain high thermodynamic efficiency.^13^ McLinden et al.^14^ determined that the choices for pure refrigerants with these desirable properties are limited, identifying only 27 pure fluids balancing the imposed safety, environmental, and technical requirements belonging to hydrofluoroolefins (HFOs), and hydrochlorofluoroolefins (HCFOs). A more flexible and potentially promising solution is the design of refrigerant blends, which are binary or ternary mixtures precisely engineered to possess the desirable properties ensuring compliance with environmental, safety, and technical requirements.^14–16^ The most critical criterion for refrigerant blends is the glide temperature, which is the difference between the dew and bubble points during phase change at constant pressure. Blends with null or low glide temperatures are preferred, which thermodynamically exhibit azeotropic or near-azeotropic phase behavior.

Thermodynamic models, based on classical or statistical thermodynamics, have provided a reliable platform for the rational design of refrigerant blends,^15,17–27^ with equations of state (EoSs) predicting thermophysical and thermodynamic properties used as inputs to screen refrigerant blends based on technical criteria and thermodynamic efficiency. In most cases, this requires predictions for phase equilibria and densities, enthalpy and entropy diagrams, and derivative and transport properties. A fundamental property often overlooked in refrigerant blend design is the surface tension, which critically impacts heat transfer in the system by influencing nucleate boiling and two-phase flow, as well as the wetting behavior on heat exchangers. Low surface tension can enhance heat transfer by reducing the superheat needed for bubble nucleation and growth. However, it can also reduce liquid surface stability which hinders heat transfer due to increased droplet formation and entrainment.^28,29^

From a fundamental aspect, azeotropic mixtures, such as those found in refrigerant blends, exhibit a unique interfacial anomaly, namely, the formation of an aneotrope, which is a nonideal surface behavior where the change in surface tension of a mixture with composition reaches a stationary point or an extremum.^30^ An existing notion suggests that azeotropic behavior and nonmonotonic density profiles are indicators for the appearance of aneotropic behavior (with relatively close compositions).^31^ Microscopically, Rowlinson and Widom^32^ highlighted that this interfacial anomaly implies a state where the bulk and surface compositions are identical. Experimentally, this was observed for mixtures close to their upper critical temperature or having pure components with similar surface tensions.^33–36^

In a series of work, Vega and co-workers,^37–40^ examined the interfacial anomalies for selected refrigerant blends between HFCs/HFOs and hydrocarbons (HCs) using molecular dynamics simulations and polar perturbed chain statistical associating fluid theory (PC-SAFT)^41^ with density gradient theory (DGT).^42,43^ They concluded that the large dissimilarity in energy scales between the polar refrigerants and nonpolar hydrocarbons is the driver for the formation of distinct aneotropic behavior at compositions relatively close to the azeotrope, observing preferential adsorption at the interface rather than passing to the liquid phase. Similarly, González-Barramuño et al.^44^ using SAFT-VR-Mie + DGT,^45^ connected the azeotropic behavior and interfacial properties of HFC + HC blends and observed a subtle interfacial reordering of absorption in the interface resulting in the interfacial anomaly. This interfacial anomaly is well established for binary refrigerant mixtures with largely dissimilar intermolecular interactions yet unexplored for blends that are dominated by polar interactions, even if at slightly different magnitudes.

In this contribution, we aim to understand the connection between azeotropy and aneotropy in refrigerant blends, particularly for mixtures of highly dipolar molecules belonging to HFCs, HFOs, and HCFOs. To this end, the role of polarity in the phase equilibria and interfacial properties of refrigerant blends is systematically investigated by integrating DGT with the soft-SAFT EoS^46,47^ (and its polar extension)^48^ to study the bulk fluid as well as microscopic and nanoscopic interfacial properties of azeotropic refrigerant mixtures.

## Methodology

### The Polar Soft-SAFT Equation of State

The polar soft-SAFT EoS used in this work,^48^ an extension of the original soft-SAFT,^46^ is written for pure fluids in terms of the residual Helmholtz free energy (*a* ^res^), as the sum of several perturbation terms accounting for different microscopic contributions,

$$a^{\mathrm{res}}=a^{\mathrm{ref}}+a^{\mathrm{chain}}+a^{\mathrm{assoc}}+a^{\mathrm{polar}}$$ 1

The reference term (*a* ^ref^) accounts for contribution of repulsive and attractive interactions between individual segments, represented by the Lennard-Jones (LJ) intermolecular potential, computed using the well-known LJ EoS developed by Johnson et al.,^49^ which describes the thermodynamic properties of this model fluid very accurately in a wide range of reduced states.^50^ The chain term (*a* ^chain^) accounts for the intramolecular interactions from the formation of chains of connected LJ segments, while the association term (*a* ^assoc^) represents the contribution of highly localized, short-range interactions such as hydrogen bonding. The expressions for *a* ^chain^ and *a* ^assoc^ are identical to other SAFT-based models, as they are based on the expressions derived by Chapman et al.^51,52^ from Wertheim’s perturbation theory.^53–56^ The radial distribution function, explicitly appearing in the chain and association terms, is based on the expressions of Johnson et al.^57,58^ for the monomer and dimer LJ fluid. Prof. Karl Johnson’s significant contributions to developing EoSs for LJ fluids are precisely why the soft-SAFT EoS is very accurate and successful. This enabled the model to have a realistic Helmholtz free energy expression, which incorporates both attractive and dispersive interactions within a single term and also appears in the radial distribution function in the chain and association terms.^59^ Lastly, the polar term (*a* ^polar^) explicitly accounts for multipolar interactions (i.e., dipolar and quadrupolar), calculated using the segment-approach expressions of Jog and Chapman,^60,61^ based on the theory of Gubbins and Twu for spherical molecules.^62,63^ The reader is referred to the original contributions for the mathematical expressions and additional details on each term.^46,48^

Applying polar soft-SAFT to pure fluids requires a molecular model, represented by a set of specific molecular parameters, that captures the key structural and energetic features of the fluid. The basic molecular parameters descriptive of nonassociating and nonpolar fluids are the chain length (*m*_*i*_), segment diameter (σ_*i*_) and dispersive energy (ε_*i*_), where the last two parameters account for the diameter and energy of interaction of the chemical groups forming the chains. In the case of associating fluids, two additional parameters are required related to the volume (κ_α–β,*i*_^HB^) and energy of association (ε_α–β,*i*_^HB^). Similarly, for fluids with polar interactions, two additional parameters are needed, namely, dipole/quadrupole moment (μ/*Q*), and fraction of polar segments (*x*_p_), which represents the proportion of the molecular volume influenced by the polar moment. These molecular parameters are typically regressed to the pure fluid’s saturated liquid density and vapor pressure data or fixed beforehand based on physical arguments.

In this work, we focus on modeling dipolar refrigerants belonging to the HFC, HFO, and HCFO families. Considering the strong dipole-moment of the refrigerants studied in this work due to the electronegativity of the fluorine atom, the *a* ^ref^, *a* ^chain^, and *a* ^polar^ terms in eq 1 are explicitly considered. Accordingly, five molecular parameters are required to describe the pure refrigerants, *m*_*i*_, σ_*i*_, ε_*i*_, and μ, *x*_p_,, where μ is fixed to available experimental values. Reliable molecular models for the studied pure refrigerants have been previously developed and validated by our group to ensure accurate description of several key properties,^64,65^ and adopted without modification in this work, with the parameters included in Table S1 in the Supporting Information for completeness.

Extending the polar soft-SAFT EoS to modeling multicomponent refrigerant mixtures is straightforward for *a* ^chain^, *a* ^assoc^, and *a* ^polar^, which are explicitly written for mixtures. Conversely, *a* ^ref^ is extended to mixtures using the generalized Lorentz–Berthelot (LB) combining rules to calculate the crossed size, σ_*ij*_, and energy, ε_*ij*_, between unlike segments,

$$\varepsilon_{ij}=\xi_{ij}\sqrt{\varepsilon_{ii}\varepsilon_{jj}}$$ 2a

$$\sigma_{ij}=\eta_{ij}\frac{\left(\sigma_{ii}+\sigma_{jj}\right)}{2}$$ 2b

where subscripts *ii* and *jj* refer to like interactions and subscript *ij* refers to unlike interactions, with η_*ij*_ and ξ_*ij*_ (η_*ij*_ = 1 – *l*_*ij*_ and ξ_*ij*_ = 1 – *k*_*ij*_ in classical EoSs) being the adjustable binary interaction parameters for crossed size and energy, respectively. The model is used in a predictive manner when η_*ij*_ and ξ_*ij*_ are set to unity. Otherwise, these parameters are regressed to the available vapor–liquid equilibrium (VLE) mixture data. Both options are applied in this work, depending on the studied system.

### Density Gradient Theory

The density gradient theory (DGT), first introduced by van der Waals^42,43^ and later revisited by Cahn and Hilliard,^66^ is widely used for computing the surface tension of pure fluids and mixtures. The theory provides a density functional for the local Helmholtz energy density of a fluid decomposed to homogeneous and inhomogeneous terms. The homogeneous terms are represented by the Helmholtz energy density of a homogeneous fluid, evaluated at a local density between the bulk densities. Conversely, the Helmholtz energy of the inhomogeneous fluid is expressed as a function of the molar density and its derivatives with respect to the space coordinates and treated as independent variables with the assumption that the density gradient is smaller than the reciprocal value of the intermolecular distance. Accordingly, the Helmholtz energy function is expanded in a Taylor series in the derivatives of the component densities with respect to the spatial coordinate normal to the interface and truncated after the second-order term, written as

$$a\left[\rho \right]=a_{0}\left(\rho \right)+\overset{2}{\underset{i=1}{\sum }}\overset{2}{\underset{j=1}{\sum }}\frac{1}{2}c_{ij}\nabla \rho_{i}\cdot \nabla \rho_{j}$$ 3

where *a*_0_(ρ)) is the Helmholtz energy of a hypothetical homogeneous fluid at the local density (composition) and *c*_*ij*_ is the influence parameter for cross-interactions between fluids *i* and *j*, obtained from the influence parameters of the pure molecules as

$$c_{ij}=\beta_{ij}\sqrt{c_{i}c_{j}}$$ 4

where *c*_*i*_ and *c*_*j*_ are the influence parameters of pure fluids *i* and *j*, respectively, regressed in this work for the first time to available pure refrigerant surface tension data for the refrigerants in Table 1. The influence parameter, which is fluid-dependent, quantifies the energetic penalty associated with variations in fluid density, which reflects the degree of spatial correlation between molecules, dictating the contribution of interactions to the overall Helmholtz energy when a fluid’s density changes across an interface. The coefficient β_*ij*_ is an adjustable parameter used to correct the deviations of the cross-influence parameter for nonideal binary mixtures, typically adjusted to mixture surface tension data. The model is used in a fully predictive manner when β_*ij*_ is fixed to unity. In this work, β_*ij*_ was fixed to unity throughout. Hence, the use of polar soft-SAFT combined with DGT to obtain interfacial properties would require the molecular parameters of soft-SAFT (i.e., *m*_*i*_, σ_*i*_, ε_*i*_, μ, *x*_p_) and the influence parameter *c*.

**Table 1:** Parameters for the Second Order Polynomial Fitting for the Temperature-Dependent Influence Parameters (*c* = *A* × *T* ^2^ + *B* × *T* + *C*), in Addition to AAD% for Surface Tension for Each Refrigerant and Data Reference Used in the Fitting Process

refrigerant	*A*	*B*	*C*	AAD%	data ref
R41	–1.14 × 10^–24^	4.38 × 10^–22^	–1.80 × 10^–20^	2.4	^79^
*R*32	–1.19 × 10^–24^	4.88 × 10^–22^	–1.32 × 10^–20^	1.5	^79^
R23	–3.46 × 10^–24^	1.36 × 10^–21^	–8.81 × 10^–20^	0.9	^79^
R161	–2.75 × 10^–24^	1.31 × 10^–21^	–8.85 × 10^–20^	3.3	^83^
R152a	–2.02 × 10^–24^	9.13 × 10^–22^	–2.79 × 10^–20^	0.9	^79^
R134a	–3.08 × 10^–24^	1.39 × 10^–21^	–5.62 × 10^–20^	1.3	^79^
R125	–4.89 × 10^–24^	2.16 × 10^–21^	–1.32 × 10^–19^	0.9	^79^
R245fa	–6.65 × 10^–24^	3.85 × 10^–21^	–3.71 × 10^–19^	1.1	^79^
R236fa	–1.81 × 10^–23^	1.08 × 10^–20^	–1.40 × 10^–18^	1.0	^79^
R227ea	–9.92 × 10^–24^	4.96 × 10^–21^	–4.20 × 10^–19^	0.9	^79^
HFO1123	–4.36 × 10^–24^	1.93 × 10^–21^	–1.52 × 10^–19^	0.4	^80^
R1243zf	–8.07 × 10^–24^	4.56 × 10^–21^	–5.24 × 10^–19^	2.7	^81^
R1234yf	–6.59 × 10^–24^	2.87 × 10^–21^	–1.61 × 10^–19^	0.7	^79^
R1234ze(E)	–7.59 × 10^–24^	3.93 × 10^–21^	–3.63 × 10^–19^	0.9	^79^
R1233zd(E)	–4.78 × 10^–24^	2.43 × 10^–21^	–8.99 × 10^–20^	0.6	^81^,^82^
R1224yd(Z)	–7.56 × 10^–24^	4.33 × 10^–21^	–4.15 × 10^–19^	1.8	^84^

The application of DGT depends on determining the density profiles across the interface. Additional details on the methodology employed to obtain these density profiles and application of DGT within the soft-SAFT formalism are included elsewhere^67–71^ and will not be repeated herein.

The surface tension (γ) of a planar interface, which is a consequence of the density profile, can be computed as

$$\gamma =\underset{i}{\sum }\underset{j}{\sum }\int_{-\infty }^{\infty }c_{ij}\;\frac{d\rho_{i}}{dz}\frac{d\rho_{j}}{dz}\;dz$$ 5

where dρ_*i*_/d*z* and dρ_*j*_/d*z* are the density profiles of molecules *i* and *j* across the interface, and *z* is the direction perpendicular to the interface.

Moreover, based on these density profiles, several nanoscopic properties can be computed including the relative surface adsorption, interfacial enrichment, and interfacial thickness. The relative surface adsorption (Γ_*i*_^(*j*)^) in a binary mixture can be calculated making use of the symmetric interface segregation as such:^72–74^

$$\Gamma_{i}^{\left(j\right)}=-\left(\rho_{i}^{l}-\rho_{i}^{v}\right)\int_{-\infty }^{\infty }\left[\frac{\rho_{j}\left(z\right)-\rho_{j}^{l}}{\rho_{j}^{l}-\rho_{j}^{v}}-\frac{\rho_{i}\left(z\right)-\rho_{i}^{l}}{\rho_{i}^{l}-\rho_{i}^{v}}\right]\;dz$$ 6

where Γ_*i*_^(*j*)^ is the relative surface adsorption of component *i* at component *j* in a binary mixture and ρ_*i*/*j*_(*z*), ρ_*i*/*j*_^l^, and ρ_*i*/*j*_^v^, are the component density profile across the interface on a nanoscopic scale and densities at saturation in the bulk liquid and vapor phases, respectively.

The interfacial enrichment *E*_*i*_, characterizing the interfacial excess at the surface, is defined as the ratio of the maximum local density of component *i* in the interfacial region, and the larger component’s *i* densities in the two bulk phases^75–77^

$$E_{i}=\frac{\max\left(\rho_{i}\left(z\right)\right)}{\max\left(\rho_{i}^{l},\rho_{i}^{v}\right)}$$ 7

Lastly, the thickness of the vapor–liquid interface is characterized in this work by the 90–10% definition for effective interfacial thickness (*L*_10_^90^), which is the distance between the points where the local density reaches 10% and 90% of the total bulk number densities^78^

$$L_{10}^{90}=z\left(\rho_{90}^{\mathrm{tot}}\right)-z\left(\rho_{10}^{\mathrm{tot}}\right)$$ 8

$$\rho_{90}^{\mathrm{tot}}=\rho_{\mathrm{tot}}^{v}+0.9\left(\rho_{\mathrm{tot}}^{l}-\rho_{\mathrm{tot}}^{v}\right)$$ 9

$$\rho_{10}^{\mathrm{tot}}=\rho_{\mathrm{tot}}^{v}+0.1\left(\rho_{\mathrm{tot}}^{l}-\rho_{\mathrm{tot}}^{v}\right)$$ 10

## Results and Discussions

### Pure Refrigerant Surface Tension Characterization

The polar soft-SAFT equation was combined with DGT to study the interfacial properties of the pure refrigerants using the reliable molecular models developed in our previous contributions,^64,65^ provided in Table S1 in the Supporting Information. The influence parameter for each refrigerant was fitted in this work to available surface tension data for the saturated liquid. Three different approaches for correlating the influence parameter were tried for selected pure refrigerants, including HFO1123, R1234yf, and R1233zd­(E): (1) a single, fixed influence parameter, fitted at an intermediate temperature along the coexistence curves (shown in Figure 1a as dashed lines), (2) a temperature-dependent influence parameter with a linear correlation (dashed-dotted line in Figure 1a), and (3) a temperature-dependent influence parameter with a second-order polynomial correlation (solid line, Figure 1a, with parameters for the second-order polynomial fitting provided in Table 1). Although the predicted surface tension trends are fairly accurate with the first approach (average absolute deviation (AAD%) < 10%) compared to experimental data,^79–82^ large deviations were observed at conditions farther from that included in the fitting (at low temperature close to the triple point and high temperature close to the critical point). Higher accuracy and better correlation to experimental trends are obtained with temperature-dependent influence parameters fitted at every reported surface tension–temperature literature data point (third approach), as shown in Figure 1a (solid lines). Irrespective of the correlation order, we have observed that the value of the influence parameter gradually decreases with increasing temperature, diminishing to zero at the critical point with the absence of a vapor–liquid interface and hence surface tension, in this region, as in Figure 1b.

**Figure 1:**
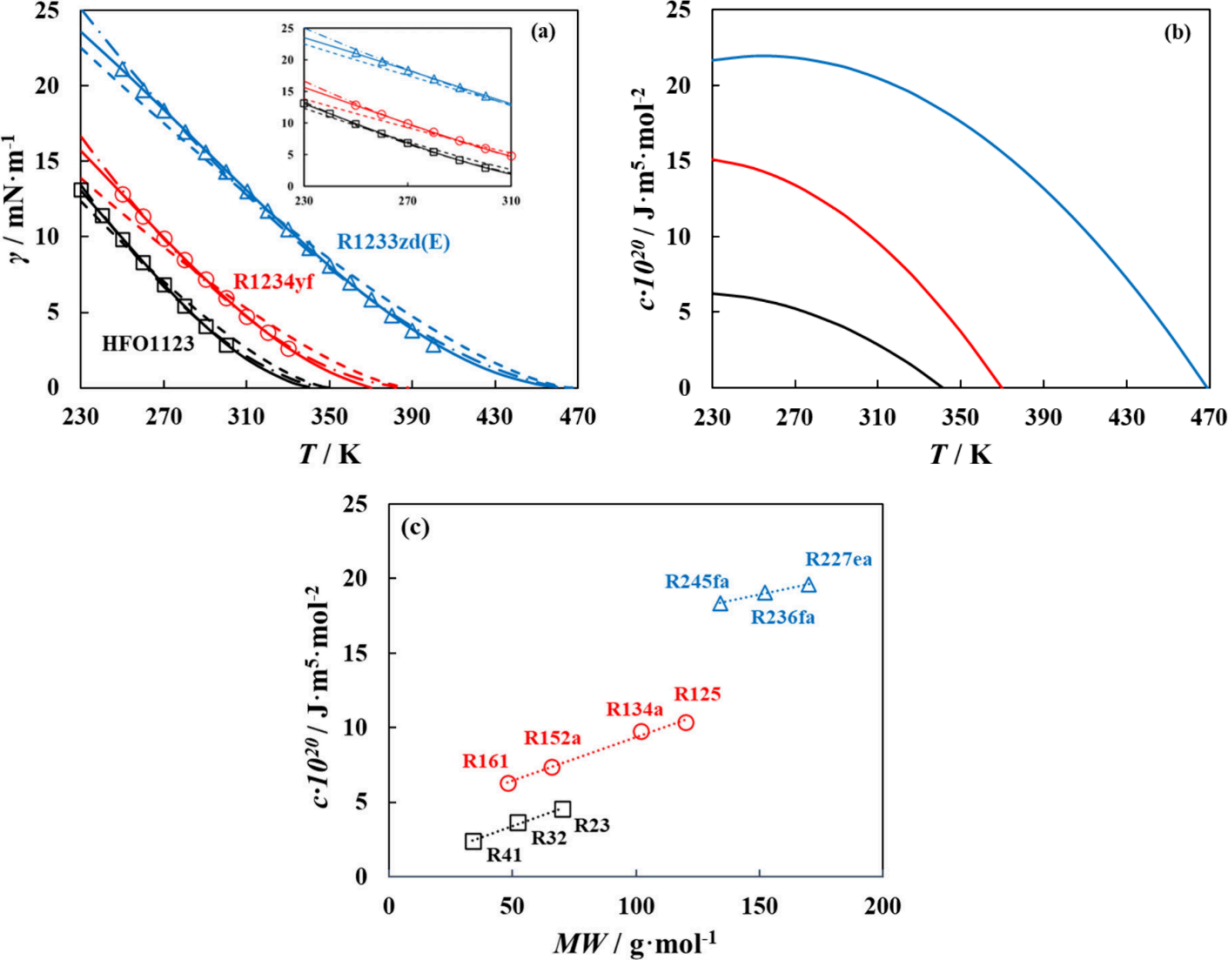
(a) Surface tension for selected refrigerants (R1233zd­(E) (blue), R1234yf (red), and HFO1123 (black)) computed from polar soft-SAFT + DGT (lines) compared to experimental data^79–82^ (symbols) using constant influence parameter (dashed lines), temperature dependent linear correlation (dashed-dotted lines), and temperature dependent second-order polynomial correlation (solid lines); inset is a zoom in for 230–310 K temperature range. (b) Temperature-dependent influence parameter trends (fitted to second-order polynomial as in Table 1) for selected refrigerants (R1233zd­(E) (blue), R1234yf (red), and HFO1123 (black)). (c) Molecular weight influence parameter trends for HFCs as a function of their molecular weight; symbols represent the value of the refrigerant’s influence parameter (using correlation in Table 1) at a given temperature (200 K for fluorinated methane and ethane families, and 270 K for fluorinated propane family), and dotted line represents the linear correlation.

The resulting set of influence parameters were correlated with temperature along the entirety of the coexistence curves for each pure refrigerant, with fitting to a second-order polynomial (average AAD = 1.6%) yielding a higher accuracy compared to a linear correlation (average AAD = 3.9%). This stringent accuracy requirement is necessary to avoid misleading results when analyzing microscopic and nanoscopic interfacial properties of refrigerant mixtures in upcoming sections. The constants for the polynomial correlation are provided in Table 1.

Although the fitting process is time-consuming, fortunately, the values of the influence parameter at a given temperature follow an increasing trend with molecular weight for HFC refrigerants belonging to the same molecular family (e.g., fluorinated methane family (R41, R32, R23), fluorinated ethane family (R152a, R134a, R125), and fluorinated propane family (R245fa, R236fa, R227ea), shown in Figure 1c). This was linearly correlated with molecular weight at selected temperatures (see Figure 1c), which facilitates predicting the influence parameters for other molecules in the same family; however it is cumbersome, as these correlations must be fitted for each temperature. Other refrigerants including HFOs and HCFOs exhibit the same trend of increasing influence parameter value at a given temperature with increasing molecular weight. However, due to the limited number of studied molecules, it was not possible to develop correlations similar to HFCs.

Depicted in Figure 2 is the description of pure refrigerant surface tension along the coexistence curves with polar soft-SAFT + DGT using the influence parameters obtained from correlations in Table 1. The agreement between modeling and experimental data highlights the ability of the model to accurately correlate the experimental trends and its suitability for describing surface tension of pure refrigerants.^79–84^

**Figure 2:**
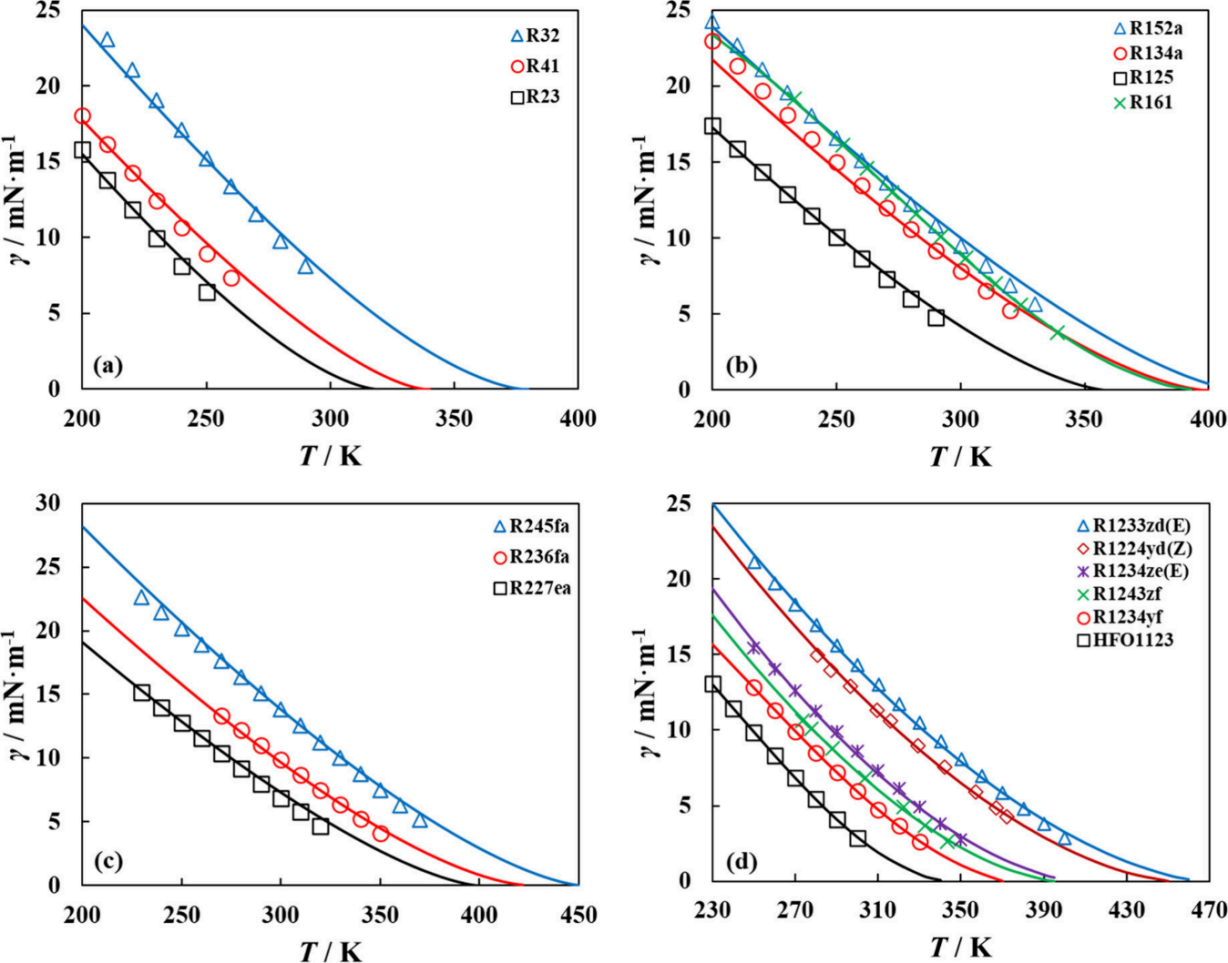
Surface tension of pure refrigerants studied in this work along their vapor–liquid coexistence curves for (a) fluorinated methane HFCs, (b) fluorinated ethane HFCs, (c) fluorinated propane HFCs, and (d) HFOs and HCFOs, with polar soft-SAFT + DGT (solid lines) using influence parameters obtained from correlations in Table 1, compared to experimental data (symbols).^79–84^

### Polar Soft-SAFT Modeling of VLE and Surface Tension of Binary Refrigerant Mixtures

Polar soft-SAFT was also used to model the phase equilibria and surface tension of binary refrigerant mixtures based on available experimental data. This is a required test to gauge the reliability of the model when extended to binary mixtures prior to the systematic study of the role of molecular effects in interfacial anomalies.

In our previous contribution,^15^ we have extensively modeled the VLE behavior of 48 binary refrigerant mixtures (e.g., HFC + HFO, HFC + HFO, HFC + HCFO), based on an abundant literature search of available experimental and simulation data, fitting the ξ_*ij*_ binary parameter when necessary, while fixing the size binary parameter (η_*ij*_) to unity in all cases. The values of these binary parameters are included in Table S2 in the Supporting Information for completeness.

The polar soft-SAFT model demonstrated its predictive power and reliability by accurately correlating experimental VLE trends with minimal to no calibration to the data. For nearly half of the studied mixtures, both binary interaction parameters (i.e., η_*ij*_ and ξ_*ij*_) were fixed to unity (see Table S2 in the Supporting Information), with accurate phase equilibria description stemming from the accurate representation of the pure refrigerants and their governing interactions. Presented in Figure 3 are a few representative mixtures including R134a + R152a, R134a + R1234ze­(E), and R1123 + R1234ze­(E), predicted from the model at different temperatures without fine-tuning to VLE data,^85–87^ encompassing several nonideal behaviors, such as formation of positive azeotropes (R134a + R152a and R134a + R1234ze­(E) mixtures) or a slight positive deviation from Raoult’s law (R1123 + R1234ze­(E)).

**Figure 3:**
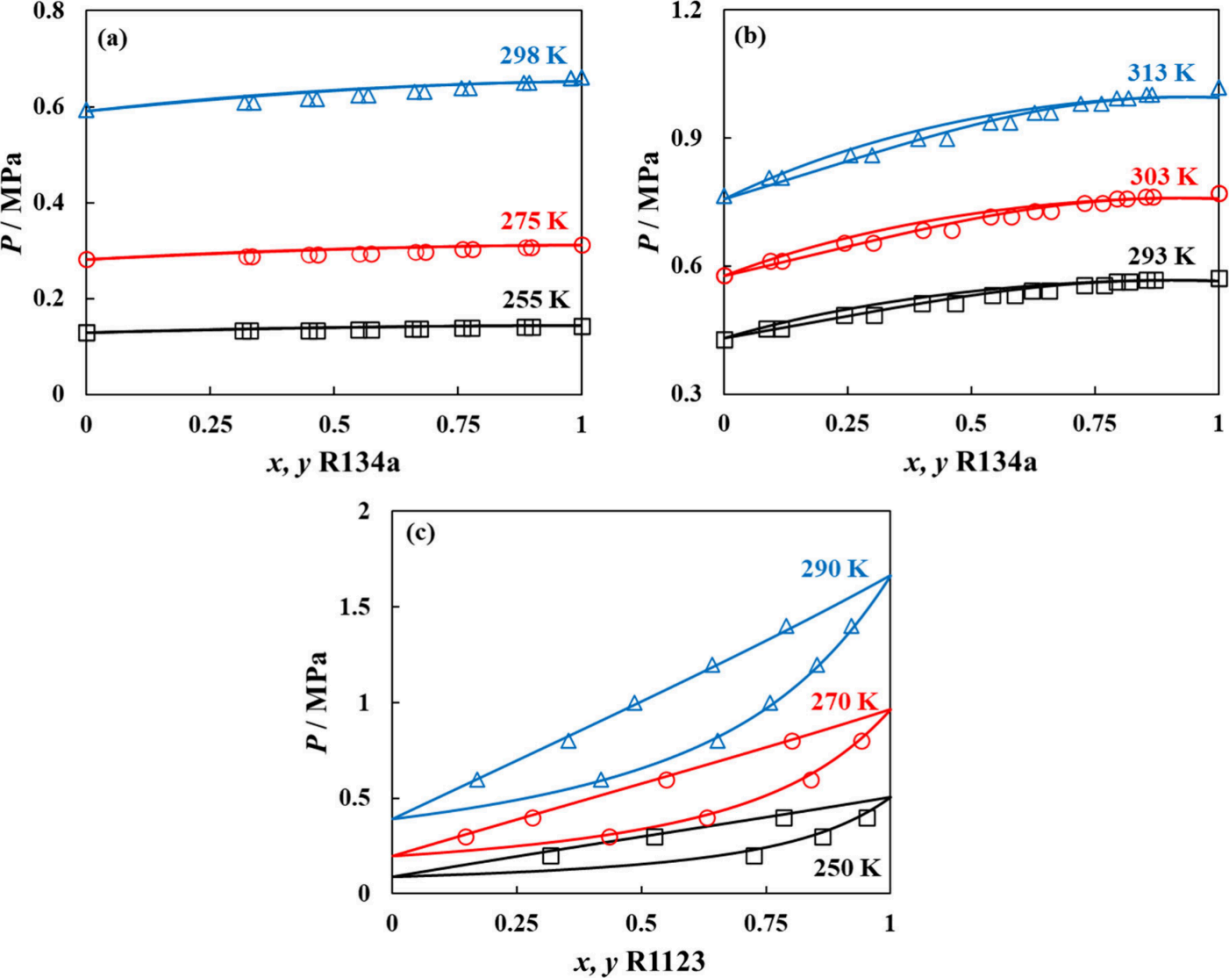
VLE behavior at different temperatures for selected binary refrigerant mixtures, including (a) R134a + R152a, (b) R134a + R1234ze­(E), and (c) R1123 + R1234ze­(E). Polar soft-SAFT predictions with unity binary parameters (solid lines) are compared to experimental data (symbols).^85–87^

For the remaining cases, minimal fine-tuning was needed to improve the accuracy of the VLE calculations, mainly to correct the variation in interaction energy scales coming from approximating the magnitude of dipolar interactions associated with *a priori* assignment of the fraction of dipolar segments (through the *x*_p_ parameter) rather than their explicit parametrization. The improved VLE description compared to available data required a temperature-independent binary energy parameter (ξ_*ij*_) for each mixture, with values close to unity in the range 0.97–1.05, which proved transferable to other mixtures with similar molecular features.

Depicted in Figure 4 are the VLE diagrams for selected binary mixtures including R32 + R134a, R32 + R1234yf, and R1234yf + R134a, comparing polar soft-SAFT calculations with (ξ_*ij*_ ≠ 1) and without (ξ_*ij*_ = 1) binary interaction parameters. Using the model in a predictive manner is reasonably accurate, slightly underestimating the mixture’s total pressure compared to experimental data (AAD = 3–8%).^88–90^ This indicates that the model interaction energy between both components is higher than required; hence, correcting the crossed interactions with binary parameters lower than unity improved the estimation of the mixture’s total pressure with average AAD < 1.5%.

**Figure 4:**
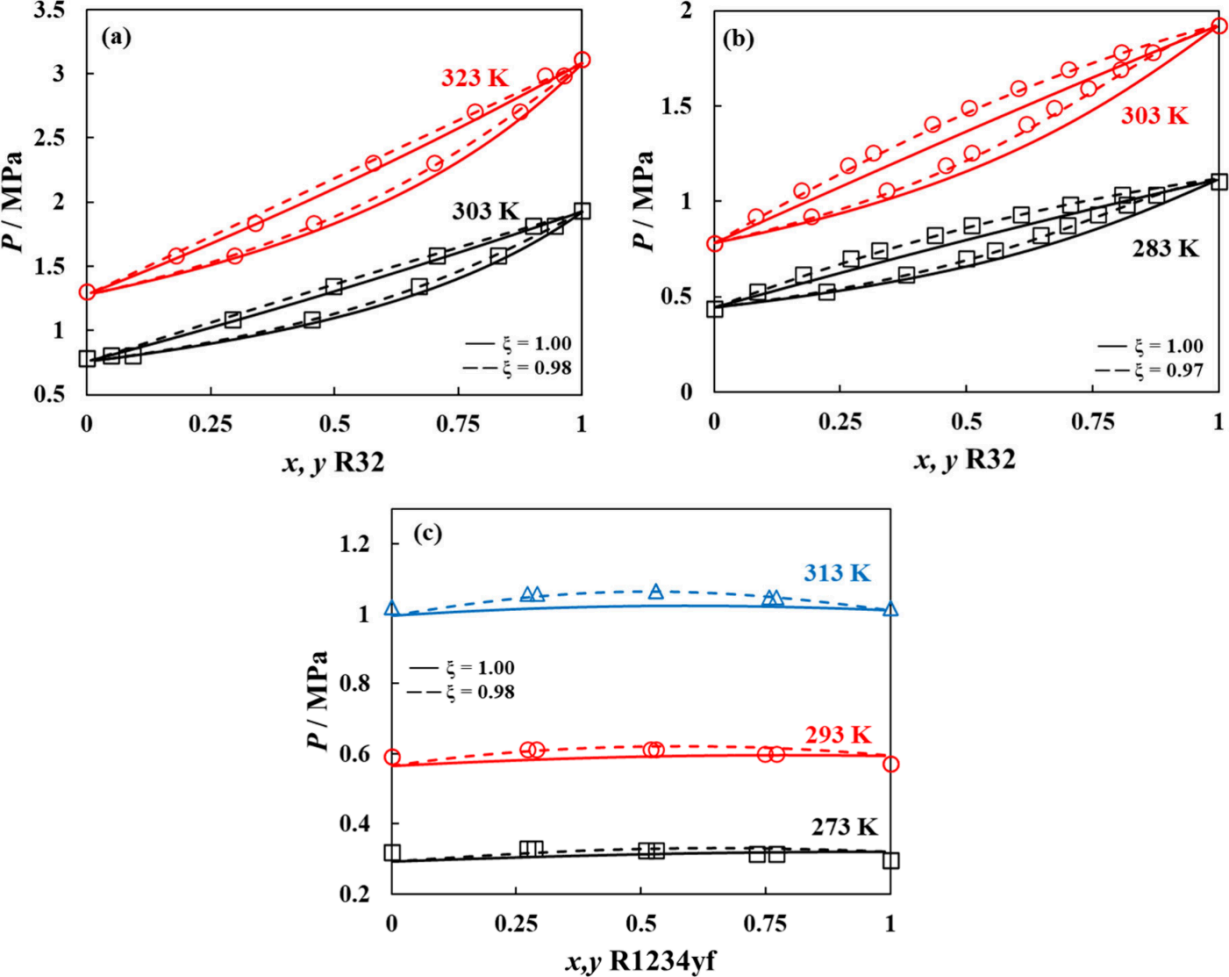
VLE behavior at different temperatures for selected binary refrigerant mixtures including (a) R32 + R134a, (b) R32 +R1234yf, and (c) R1234yf + R134a. Polar soft-SAFT predictions with unity binary parameters (solid lines) and with adjusted ξ_*ij*_ binary parameter (dashed lines) are compared to experimental data (symbols).^88–90^

Accurate description of phase equilibria is crucial for the precise modeling of interfacial properties in binary mixtures, as the latter arises from the differing interactions at the interface, which requires an exact knowledge of the compositions and properties of the coexisting phases. Hence, an inaccurate representation of phase equilibria would lead to erroneous predictions and conclusions about interfacial properties. The preceding results proved the accuracy of polar soft-SAFT in modeling the bulk fluid and its thermodynamic properties used as inputs for computing interfacial properties using DGT. The reliability of predicting the surface tension of a binary mixture is highlighted in Figure 5 for selected mixtures based on available experimental data including mixtures of HFC + HFC and HFC + HFO. The modeling results agree well with the experimental data,^91–96^ with average deviation less than 2% for all studied mixtures, albeit slight underestimation for some mixtures (R32 + R1234yf and R134a + R1234yf). The results are satisfactory considering they are purely predictive, only requiring the influence parameter of the pure refrigerant, aside from an accurate description of the phase envelope as well as the free energy in the metastable and unstable region, capable of predicting extreme phenomena such as the formation of an aneotrope for the R32 + R1234yf mixture (seeFigure [Fig fig5]d).

**Figure 5:**
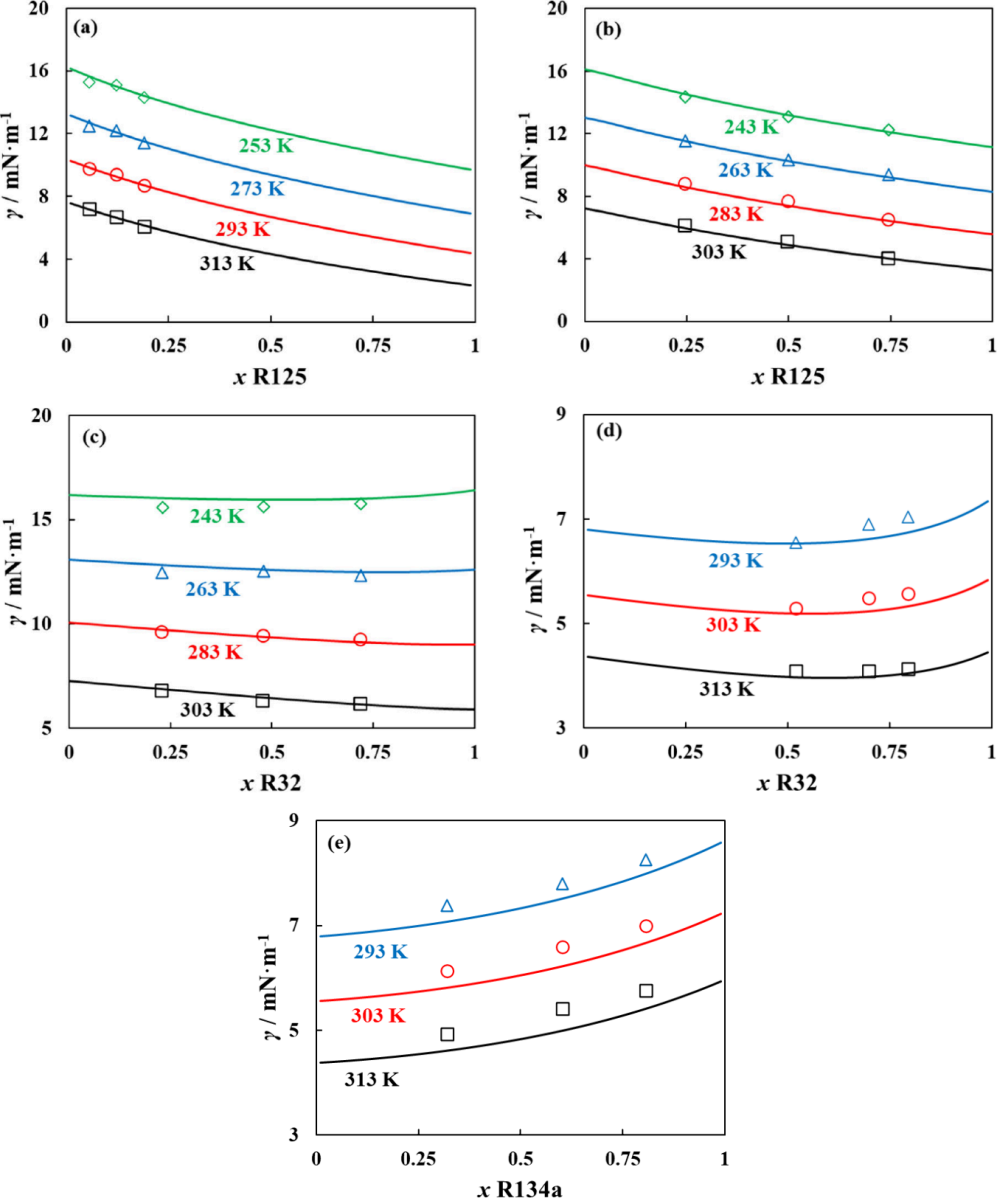
Predicted surface tension of refrigerant binary mixtures including (a) R125 + R152a, (b) R125 + R134a, (c) R32 + R134a, (d) R32 + R1234yf, and (e) R134a + R1234yf. Polar soft-SAFT + DGT predictions (solid lines) are compared to experimental data (symbols).^91–96^

### Effect of Polar Interactions on Phase Equilibria and Surface Tension of Refrigerant Binary Mixtures

With the reliability of polar soft-SAFT and its DGT extension validated for pure refrigerants and binary mixtures, we set out to elucidate the impact of polarity, in addition to other molecular features, on the phase equilibria and interfacial properties of selected refrigerant mixtures. We have opted to design these systems considering R134a as the main component, given its widespread industrial usage, while changing the second component to those listed in Table S1 in the Supporting Information. All calculations have been performed at a fixed temperature of 298 K, which is far from R134a’s critical temperature. The binary interaction parameters listed in Table S2 were used for the mixtures with R134a, except for the systems with R41, R161, HFO1123, R1233zd­(E), and R1224yd­(Z), where both binary parameters were fixed to unity (effectively using the model in a predictive manner) due to the absence of experimental data to check the adequacy of the phase equilibria calculations. This is a valid assumption considering the minimal fine-tuning needed to improve the accuracy of modeling phase envelopes for mixtures with R134a (i.e., 1 > ξ_*ij*_ > 0.98). We have checked predictions using polar soft-SAFT by transferring the binary energy parameter from similar refrigerants, without noticeable variation in the VLE behavior and total pressure magnitude.

Depicted in Figure 6 are the VLE curve predictions for selected binary mixtures with R134a at 298 K, focusing on variability in the number of carbons, degree of fluorination, and halogen type to gain insights into the role of variability in molecular features on the change in phase behavior. From a molecular point of view, negative deviation from Raoult’s law indicates the presence of strong attractive interactions between unlike molecules in the mixture, and the opposite is true for positive deviations. Some R134a mixtures exhibit nearly ideal behavior such as R134a + R245fa. Others exhibit slight negative deviation from ideality observed for R134a with R41 and HFO1123) or positive deviation as with R134a mixtures with R32 and R125. In other instances, the positive deviations are more prominent with the formation of azeotropes (R134a with R161, R152a (near azeotropic), R1243zf, and R1234ze­(E)) or wider VLE curves over large ranges of pressure (R134a + R1233zd­(E)).

**Figure 6:**
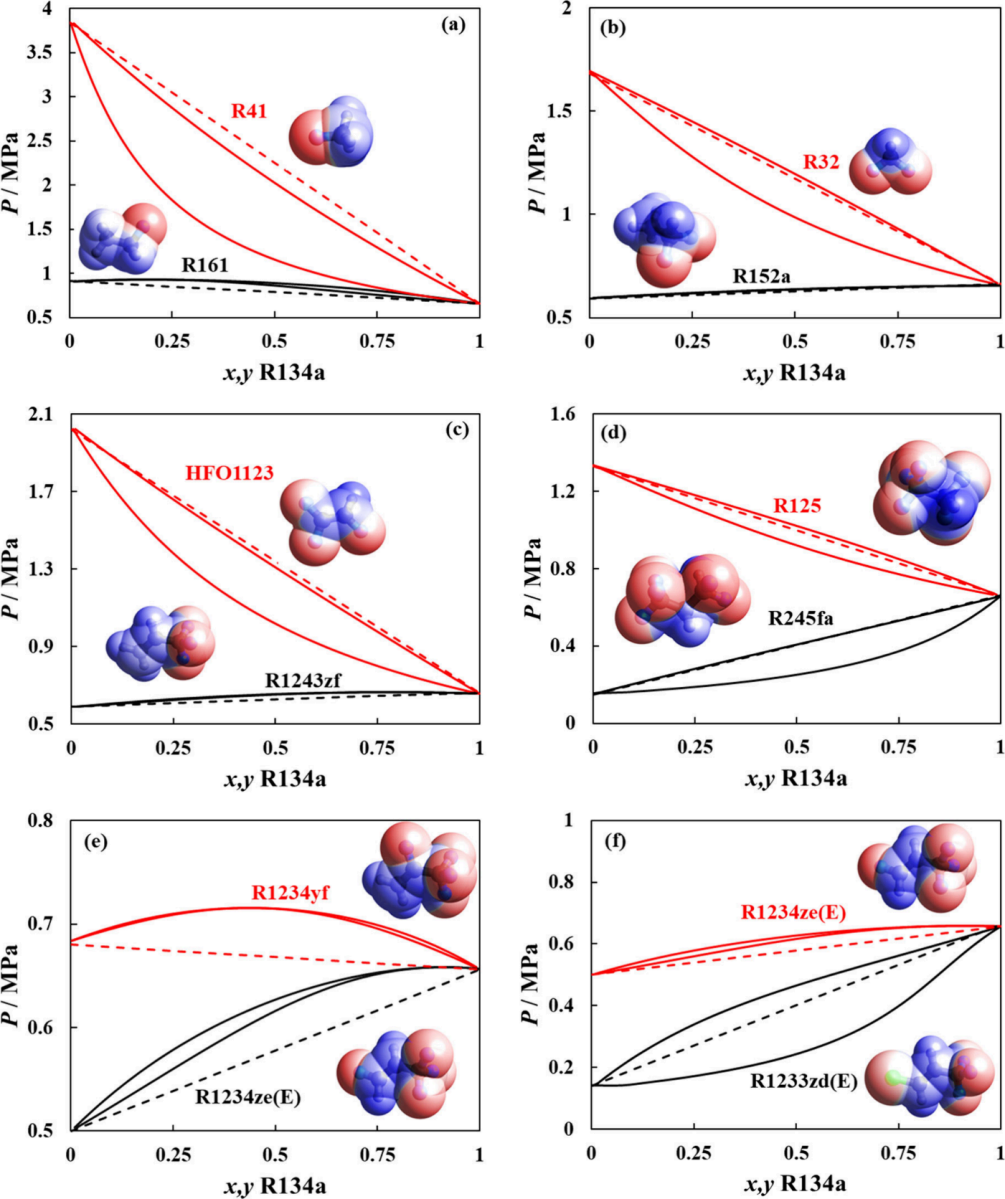
Predicted VLE envelopes at 298 K for binary mixtures between R134a and (a) R41/R161 (b) R32/R152a, (c) HFO1123/R1243zf, (d) R125/R245fa, (e) R1234yf/R1234ze­(E), and (f) R1234ze­(E)/R1233zd­(E). Polar soft-SAFT predictions using binary parameters in Table S2 (solid lines) and Raoult’s law ideality (dashed lines).

For molecules with the same degree of fluorination (see Figure 6a–c), increasing the length of the carbon chain (focusing on HFCs from fluorinated methane R41/R32 to fluorinated ethane R161/R152a in Figure 6a,b) increases the extent of positive deviation from Raoult’s law, with the appearance of azeotropic mixtures (or near azeotropic for R134a + R152a). As the chain length of the second component increases, its overall molecular volume also increases, leading to stronger dispersive interactions within its carbon chain. Additionally, these larger molecules exhibit higher polarities compared to R134a, though not scaled proportionally, resulting in less conducive structures and charge distribution for strong interactions with R134a. This trend also applies to HFOs, with the transition from fluorinated ethene HFO1123 to fluorinated propene R1243zf, shifting the VLE curve from negative to positive deviation from ideal behavior as seen in Figure 6c. That said, this trend does not hold for R134a + R125 and R134a + R245fa (see Figure 6d), as the latter mixture exhibits a near ideal VLE curve compared with the slight positive deviation for R134a + R125. This near ideal behavior for R134a + R245fa can be explained by the components’ structural resemblance with fluorine atoms distributed on both ends of the chain resulting in relatively similar charge distribution which allows R134a’s higher polarity to compensate for R245fa’s larger dispersive interactions.

When it comes to the degree of fluorination for molecules within the same homologous series, the observed trend is that increasing the degree of fluorination is accompanied by a reduced number of deviations from ideal behavior. For example, the mixture shifted from a notable negative deviation with R41 to a slight positive deviation with R32 (see Figure 6a). Similarly, transitioning from R161 to R152a resulted in a change from a notable to a slight positive deviation (see Figure 6b). The increasing number of fluorine atoms increases the polarity of the molecule to levels similar to that for R134a, leading to relatively similar energy scales and near ideal behavior as that with R134a + R245fa (see Figure 6d).

This also depends on the position of the fluorine atoms and their impact on the overall charge distribution of the molecule, as is the case for mixtures with the structural isomers R1234yf and R1234ze­(E) in Figure 6e. The shift in fluorine atom from the second carbon to the third carbon along the double bond, from a central position to at the chain end, makes the charge distribution more homogeneous for R1234ze­(E) compared to R1234yf. As a result, the electron withdrawing effects of the −CF_3_ group are diminished, reducing the repulsive interactions and the extent of positive deviation from ideality. The same effect is also seen when changing the halogen atom from fluorine to the less electronegative chlorine atom, with the positive deviation changing from the azeotropic R134a + R1234ze­(E) mixture to a wider VLE curve for R134a + R1233zd­(E) as shown in Figure 6f.

The surface tension for binary mixtures with R134a was also predicted at 298 K with the DGT extension to polar soft-SAFT, obtained directly from the influence parameters for pure refrigerants shown in Table 1. The change in surface tension behavior with varying the second component in the mixtures with R134a seems rather regular for most cases without anomalous tendencies, despite the nonideal VLE characteristics for the mixtures, as shown in Figure 7. For mixtures with near ideal behavior or slight nonideality, the change in the mixture’s surface tension is monotonous and regular, gradually changing the surface tension to that of pure R134a with the latter’s increasing content in the mixture, as the case for R134a + R32, and R134 + R245fa in Figure 7a,b. A highly nonideal VLE curve does not necessarily translate to an anomaly in surface tension. For example, a monotonous regular reduction in surface tension with increasing R134a content is observed for the nearly azeotropic R134a + R152a (see Figure 7a) and the azeotropic R134a + R1234yf and R134a + R1233zd­(E) with a positive VLE deviation (see Figure 7b). From these examples, we captured an aneotropic behavior only for the azeotropic R134a + R1234ze­(E).

**Figure 7:**
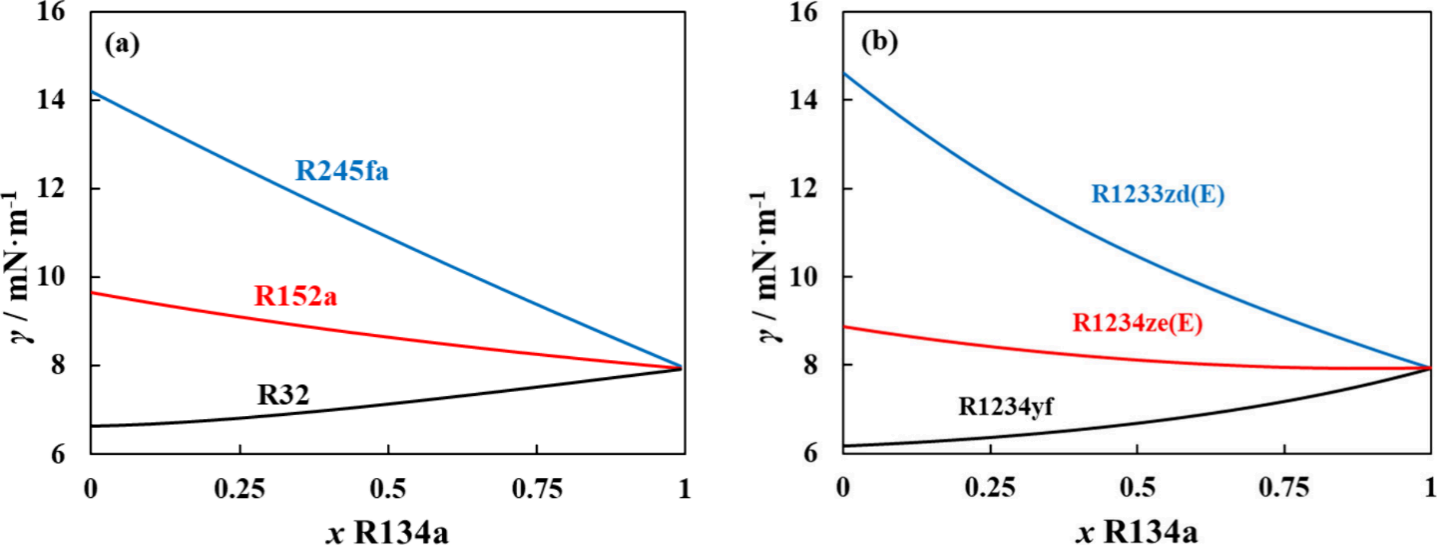
Predicted surface tension at 298 K for binary mixtures between R134a and (a) R245fa/R152a/R32 or (b) R1233zd­(E)/R1234ze­(E)/R1234yf. Polar soft-SAFT + DGT predictions (solid lines).

**Table 2:** Composition for the Azeotrope and Aneotrope (if Any) for the Binary Mixtures in Figure 8

	Azeotrope		Aneotrope	
Mixture	*P* (MPa)	*x*_R134a_	γ (mN·m^–1^)	*x*_R134a_
R134a + R161	0.932	0.192	7.806	0.731
R134a + R152a				
R134a + R227ea			7.022	0.177
R134a + R1243zf	0.664	0.780	7.505	0.368
R134a + R1234yf	0.716	0.435		
R134a + R1234ze(E)	0.658	0.911	7.918	0.892

### Microscopic and Nanoscopic Interfacial Anomalies in Azeotropic Refrigerant Mixtures

In this section, we set out to provide deeper insight into the appearance of aneotropic behavior for azeotropic refrigerant mixtures. From 15 mixtures with R134a predicted from polar soft-SAFT at 298 K, the formation of positive azeotropes with distinct pressure maxima was captured for only four mixtures with R134a, including R161, R1243zf, R1234yf, and R1234ze­(E), all of them depicted in Figure 8a. These four mixtures have relatively narrow boiling phase behavior (i.e., the liquid and vapor phases have similar compositions). Interestingly, mixtures with R152a and R227ea in Figure 8b present near azeotropic behavior (near identical liquid and vapor compositions) when the R134a composition is higher than 75% but with no pressure maxima observed in the bulk phase (the thermodynamic condition for azeotropy). For these mixtures, aneotropic behavior was detected, except for the azeotropic R134a + R1234yf and near azeotropic R134a + R152a (see Figure 8c,d). Remarkably, an aneotropic behavior was captured for the near azeotropic R134a + R227ea mixture, indicating that the azeotropic tendency was sufficiently strong to create an anomaly at the interface, which has not been reported before in the literature for nonazeotropic mixtures, except for the work of Fouad et al.^39^ for R32 + R1234yf. The case of an aneotrope for the nonazeotropic R134a + R227ea mixture and the absence of interfacial anomaly for the azeotropic R134a + R1234yf mixture clearly demonstrate that the presence of aneotropy will not necessarily be accompanied by a distinct azeotropic behavior in the thermodynamic definition and vice versa.

**Figure 8:**
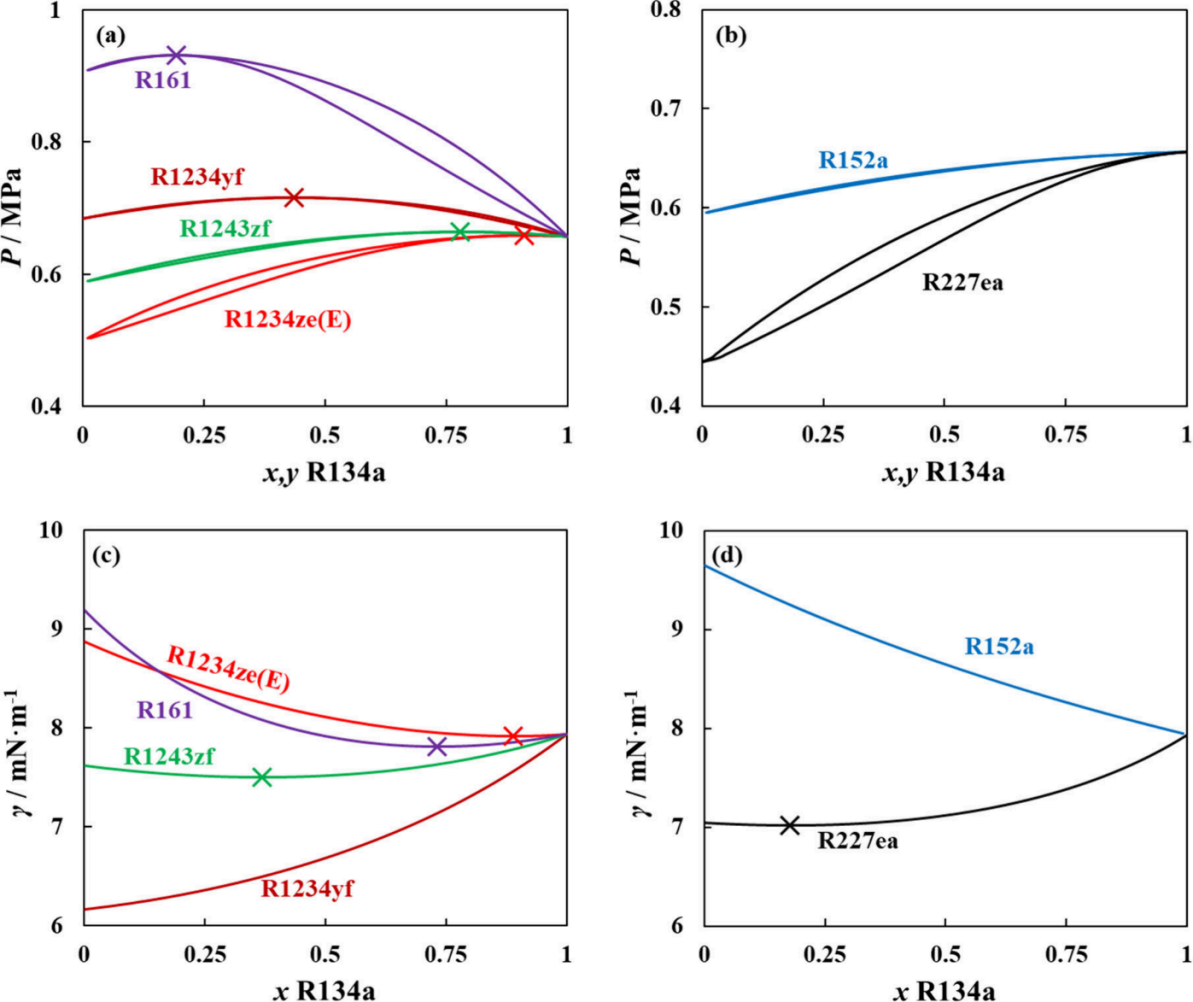
Predicted VLE curves (top) and surface tension (bottom) for binary mixtures with R134a for (a, c) azeotropic mixtures and (b, d) near azeotropic mixtures. Polar soft-SAFT + DGT predictions (solid lines) performed at 298 K. The symbol (×) denotes the composition of the azeotrope on the VLE curves in (a) and the aneotrope on the surface tension curve in (c) and (d).

For the mixtures exhibiting interfacial anomaly, the aneotrope composition does not coincide with the azeotrope composition provided in Table 2, except for the R134a + R1234ze­(E) mixture, which is not necessarily the case reported in literature focusing on mixtures of polar and nonpolar refrigerants.^38,44^ However, the aneotrope composition for the mixtures studied herein exhibits a trend that nearly follows (*x*_an_ = 1 – *x*_az_), which might be related to the governing dipolar interactions in the studied mixtures.

The density profiles for the binary mixtures exhibiting aneotropic behavior were analyzed at the composition of the aneotrope and azeotrope (if not the same) and are presented in Figure 9. For all cases, starting in the bulk vapor phase, the component density for both R134a and the second component in the mixture increase monotonously toward the liquid bulk phase, with no apparent accumulation of either component at the interface, which can be related to the relative similarity in polarity scale between both components. This type of structural behavior is not unusual for ideal and positive azeotropic mixtures,^97^ though several studies on aneotropic behavior observed a maximum in the density profile,^38,40,44^ absent from those studied in this work. The monotonic behavior of the density profiles suggests a lack of strong surface activity from either component—that is, neither component exhibits significant interfacial enrichment compared to the bulk. This observation is not directly related to the aneotropic behavior but rather reflects the uniformity of the density profiles. A possible enrichment (i.e., nonmonotonicity of a density profile at the interface) is related to the partition coefficient (*K* = *x*/*y*). Large partition coefficients, as with wide-boiling phase behavior, are known to favor enrichment.^98^ The results obtained here are in line with these expectations. The appearance of distinct enrichment at the interface or surface excess as those reported in the literature is mainly from the large dissimilarity in energy scales and/or weak cross interactions (favoring large partition coefficients) for the binary refrigerant mixtures,^76^ typically found between highly polar refrigerants or solvents and nonpolar hydrocarbons, resulting in a nearly convex surface tension curve with a distinct minimum.^37–40,74,99,101^

**Figure 9:**
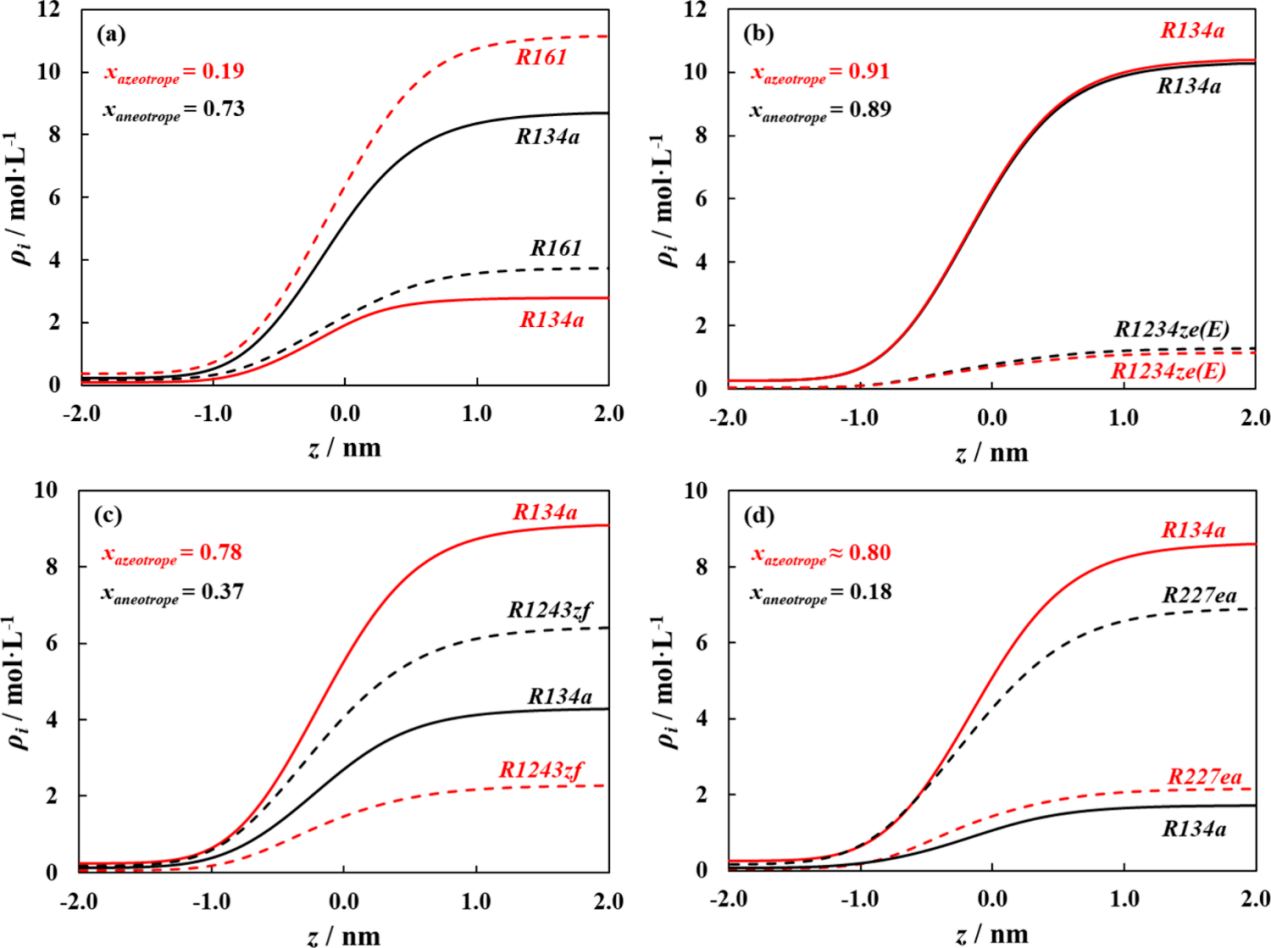
Predicted component density profiles for aneotropic binary mixtures (a) R134a + R161, (b) R134a + R1234ze­(E), (c) R134a + R1243zf, and (d) R134a + R227ea, predicted at the azeotropic composition (red lines) and aneotropic composition (black lines). Density profile for R134a (solid lines), and second component (dashed lines).

A more thermodynamically rigorous quantification of surface excess is the relative adsorption, though influenced by the monotonicity of density profiles, which provides a direct link between macroscopic interfacial anomalies and nanoscopic relative adsorption at the interface. The relative adsorption was predicted for the studied mixtures and plotted in Figure 10. Notice that the aneotropic composition consistently matches the composition at which the relative adsorption at the interface reaches zero, a state in which the bulk and mean surface compositions are identical. This is absent for mixtures not exhibiting aneotropic behavior (R134a + R152a and R134a + R1234yf in Figure 10b). For aneotropic mixtures, R134a presents slightly higher surface activity than the second component for all cases, with its preferential adsorption at the interface reducing the surface tension to lower than that of both pure components in the mixture. The higher surface activity of R134a is subtle, though with a monotonic density profile. A closer look at the density profile of the aneotropic composition shows a slight shift in the density profile of R134a in the −*z* direction with respect to the second component. This indicates that R134a adsorbs at the interface, in very small quantities, even in the absence of enrichment, yet sufficient ones to induce anomalous interfacial behavior.

**Figure 10:**
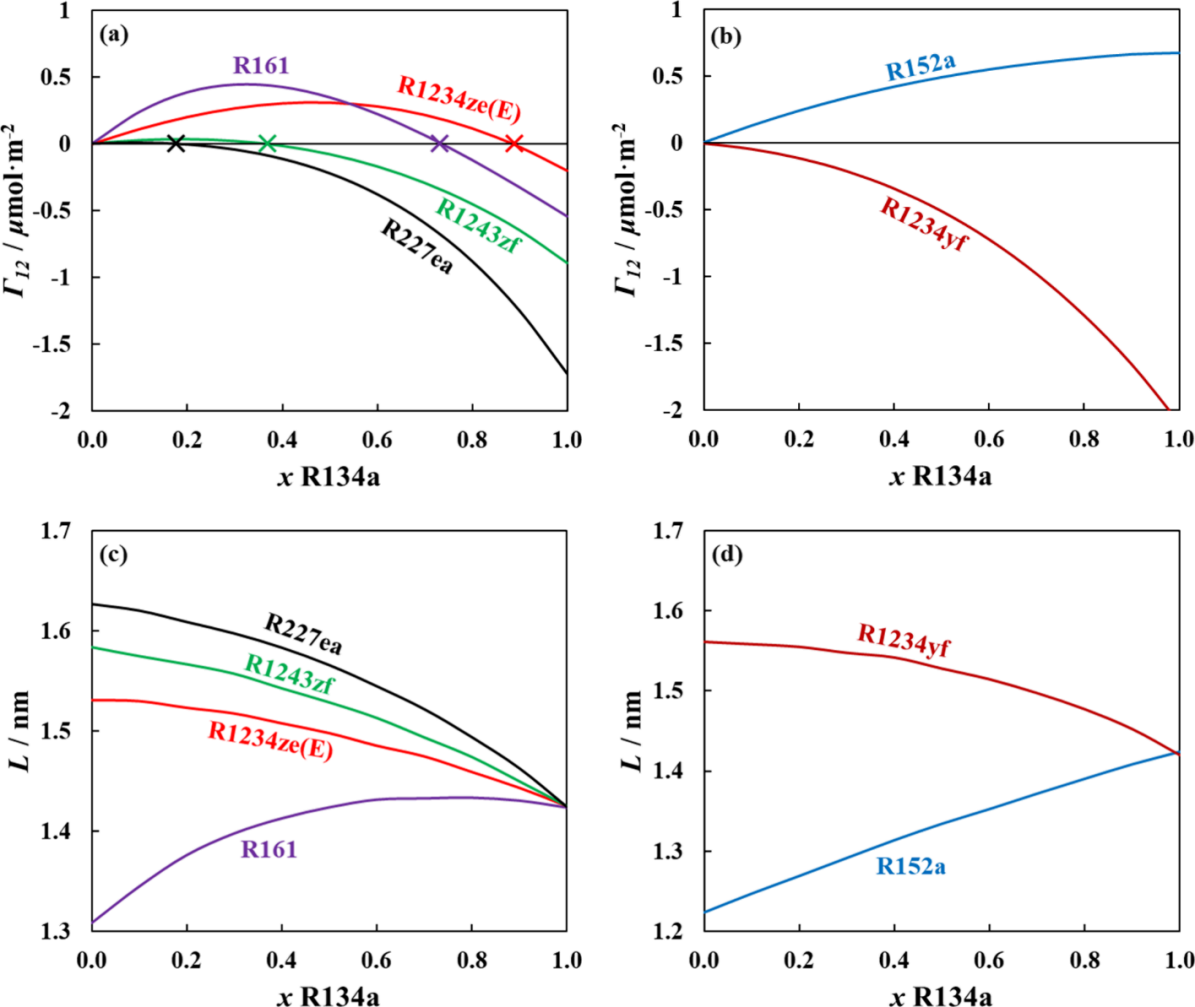
Predicted nanoscopic interfacial properties for adsorption at the interface (top) and interfacial thickness (bottom) for binary mixtures with R134a for (a, c) mixtures with aneotropic behavior and (b, d) mixtures without aneotropic behavior. Polar soft-SAFT + DGT predictions (solid lines) performed at 298 K. The symbol (×) denotes the composition of the aneotrope.

Increasing the concentration of R134a in the mixtures results in a positive relative adsorption (magnitude higher for mixtures where R134a is the high boiling point component), where R134a’s mean surface concentration is higher than that in the bulk phases, being responsible for the reduction of the mixtures’ surface tension. This continues until the interface is saturated with R134a, where no dramatic change in surface tension is observed (nil adsorption), after which, the second component is repelled from the interface with negative adsorption, and the mixture’s surface tension approaches that of pure R134a. Notice that the nil adsorption point with aneotropy occurs at low R134a concentrations for mixtures with R227ea and R1243zf (high R134a surface activity for these mixtures), compared to higher R134a contents for mixtures with R161 and R1234ze­(E) (low R134a surface activity). This is mainly related to intermolecular interactions, with the low aneotropic composition for mixtures where R134a is the component with low boiling point, suggesting stronger R134a–R134a interactions than unlike interactions with the highly repulsive R227ea or R1243zf, driving R134a to segregate at the interface to increase its preferential like interactions. For the case of high aneotropic composition with low boiling point components, competitive adsorption at the interface between R134a and R161 or R1234ze­(E) might be present; otherwise, the unlike interactions are stronger than like interactions, requiring high R134a content to become the dominant species to segregate at the interface and increase R134a–R134a interactions.

Polar soft-SAFT + DGT predicted no enrichment (*E* = 1) as a function of R134a composition for its aneotropic mixtures at 298 K (not plotted here), which supports the absence of distinct maxima in the density profiles. This is in line with results reported in the literature for different azeotropic/aneotropic simple fluid mixtures.^102^ Although both relative adsorption and enrichment describe the surface excess, they still do not contain the same information. Preferential adsorption might still occur without enrichment, especially when the densities of the two components are slightly shifted with respect to one another.^97,102^ Stephan and Hasse^98^ noted that strong enrichment at the interface is typically found for systems with a supercritical component or large variance in boiling points between the two components and positive azeotrope mixtures with large partition coefficients, which is not the case for the mixtures with R134a studied here.

In terms of the thickness of the interface (see Figure 10c,d), a decreasing trend with an increasing R134a content is observed for all mixtures, except those with R161 and R152a. With higher R134a content, the molecules at the vapor–liquid boundary are more strongly held to the interface with their respective phases, as these are more dominated by R134a, leading to a sharper and less diffuse transition zone between the two bulk phases. This is not seen with R152a and R161, as both components have similar structural and energetic characteristics to R134a, with relatively similar scale of unlike and like interactions. The qualitative trends for the interfacial thickness obtained for the refrigerant mixtures in this work are also in line with results reported in the literature for simple azeotropic/aneotropic mixtures.^102^

## Conclusions

In this work, polar soft-SAFT + DGT was systematically employed to determine the molecular origin of the interfacial anomalies in binary refrigerant blends with dipolar nature, elucidating the connection between azeotropy and aneotropy in these mixtures. Relying on reliable molecular models for 16 pure refrigerants belonging to HFCs, HFOs, and HCFOs, the surface tension calculations were validated by using available experimental surface tension data. Temperature-dependent influence parameters for the pure refrigerants were regressed to a second-order polynomial to enable describing the surface tension along the coexistence curves from triple to critical points. The model proved highly accurate and reliable with accurate representation of binary mixture phase equilibria and surface tension to existing systems with minimal fine-tuning to available binary mixture phase equilibrium data.

After validating the model, we systematically predicted the VLE behavior and surface tension of binary mixtures with R134a (as it has an intermediate dipole moment compared with the remaining molecules). The predictions demonstrate a variety of VLE behavior from nearly ideal to significantly nonideal systems exhibiting positive and negative deviations from Raoult’s law and the formation of azeotropes, profoundly impacted by variations in molecular features relative to R134a. Increasing the carbon chain length in the second component generally led to a greater extent of positive deviation and the appearance of azeotropes. Conversely, increasing the degree of fluorination tended to reduce deviations from ideal behavior, as the molecule’s polarity became more akin to that of R134a.

For mixtures exhibiting azeotropic or near azeotropic behavior, microscopic and nanoscopic interfacial properties were evaluated, including surface tension, density profiles, adsorption at the interface, enrichment, and interfacial thickness. It was determined that the presence of an aneotrope does not necessarily coincide with a distinct azeotropic point, and vice versa, highlighting the complex interplay of molecular interactions at the interface. For the mixtures exhibiting aneotropic behavior, the aneotrope composition consistently aligned with the point where the relative surface adsorption reached zero rather than at the azeotropic composition, signifying a state in which the bulk and surface compositions are identical. Additionally, for all examined cases, a distinct interfacial enrichment was absent, which can be attributed to the specific features of the underlying phase behavior such as narrow-boiling positive azeotrope, unlike cases for polar + nonpolar mixtures reported in the literature. This suggests that, while preferential adsorption of R134a at the interface can occur, it is often subtle and does not lead to a significant accumulation or “excess” at the interface. Despite this absence of strong enrichment, R134a often exhibits slightly higher surface activity, subtly influencing the surface tension. This underlines the fact that enrichment and relative adsorption are properties expressing different things, albeit being closely related. We emphasize that this is a purely predictive study based on thermodynamic modeling using parameters validated against experimental data when available. Future work will focus on validating the interfacial behavior through experimental measurements and molecular dynamics simulations.

Nevertheless, this work provides a fundamental understanding of how molecular features and polarity govern phase equilibria and interfacial properties, offering crucial insights into developing refrigerant blends that possess optimal thermophysical properties, for enhanced heat transfer and overall system efficiency. It also shows the need to have accurate molecular-based equations of state for reliable predictions, highlighting the pioneering work of Professor K. Johnson on developing the Lennard-Jones SAFT equation and its impact on the field.

## Supplementary Material



## Data Availability

Data will be made available on request.

## Notes

Polar soft-SAFT molecular parameters for pure refrigerants studied in this work and energy binary parameters (ξ_*ij*_) for binary refrigerant mixtures of HFC + HFC, HFC + HFO, and HFC + HCFO (PDF)
