# Supplementary material for: Molecular Origin of Interfacial Anomalies in Azeotropic Refrigerant Mixtures
Source: J Phys Chem C Nanomater Interfaces. 2025 Aug 4;129(32):14622–37. doi: 10.1021/acs.jpcc.5c04188 (PMC12359403; doi:10.1021/acs.jpcc.5c04188)
Supplement: Supplementary file 1 [file jp5c04188_si_001.pdf]

Supplementary Material

Molecular Origin of Interfacial Anomalies in Azeotropic Refrigerants Mixtures

Ismail I.I. Alkhatib[1*], Carlos G. Albà[2,3], Yuting Li[1], Simon Stephan[4], Fèlix Llovell[2], Lourdes F. Vega[1*]

[1]Research and Innovation Center on CO_2 and Hydrogen (RICH Center) and Chemical and Petroleum Engineering Department, Khalifa University of Science and Technology, PO Box 127788, Abu Dhabi, United Arab Emirates.
[2]Department of Chemical Engineering, ETSEQ, Universitat Rovira i Virgili (URV), Campus Sescelades, Av. Països Catalans 26, 43007, Tarragona, Spain.
[3]GasN2, Avda. Diagonal 579, Barcelona, Spain
[4]Molecular Thermodynamics Group (MTG), RPTU Kaiserslautern, Erwin-Schrodinger-Strasse 44, 67663, Kaiserslautern, Germany

[*] Corresponding authors *E-mail addresses*: lourdes.vega@ku.ac.ae; Ismail.alkhatib@ku.ac.ae

Table S1. Polar soft-SAFT molecular parameters for pure refrigerants studied in this work, transferred from previous works. [1,2] Molecular structures are drawn using PubChem Sketcher. Notice the R prefix is designated based on ASHRAE classification otherwise listed as HFO.

Refrigerants	Structure	m [-]	σ [Å]	ε/k_B [K]	$\mu\cdot10^{-30}$ [C·m]	x_p
	**HFC**					
Fluoromethane (R41)	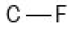	1.371	3.400	180.3	6.17	0.50
Difluoromethane (R32)	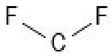	1.376	3.506	164.5	6.59	0.75
Trifluoromethane (R23)	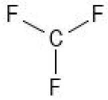	1.397	3.610	147.9	5.50	0.90
1-fluoroethane (R161)	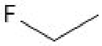	1.577	3.693	232.3	6.47	0.33
1,1-difluoroethane (R152a)	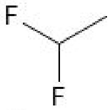	1.662	3.754	202.3	7.55	0.50
1,1,1,2-tetrafluoroethane (R134a)	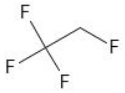	1.813	3.770	169.5	6.86	0.70
1,1,1,2,2-pentafluoroethane (R125)	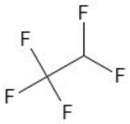	1.887	3.790	165.1	5.21	0.90
1,1,1,3,3-pentafluoropropane (R245fa)	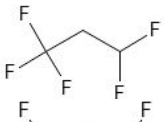	2.479	3.675	197.1	5.17	0.80
1,1,1,3,33-hexafluoropropane (R236fa)	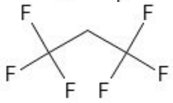	2.056	4.012	172.4	6.61	0.90
1,1,1,2,3,3-heptafluoropropane (R227ea)	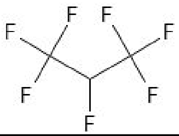	2.131	4.033	190.7	4.86	1.00

**HFO**						
1,1,2-trifluoroethene (HFO1123)	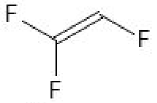	1.527	3.760	175.3	5.74	0.80
3,3,3-trifluoroprop-1-ene (R1243zf)	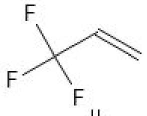	1.904	3.880	170.0	8.17	0.50
2,3,3,3-tetrafluoroprop-1-ene (R1234yf)	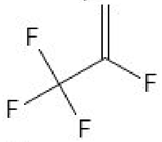	1.740	4.082	191.6	6.71	0.70
trans-1,3,3,3-tetrafluoroprop-1-ene (R1234ze(E))	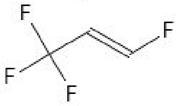	2.044	3.821	204.0	4.80	0.75
**HCFO**						
trans-1-chloro-3,3,3-trifluoroprop-1-ene (R1233zd(E))	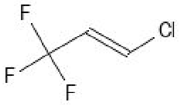	2.331	3.819	232.6	3.81	0.80
cis-1-chloro-2,3,3,3-tetrafluoroprop-1-ene (R1224yd(Z))	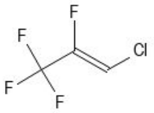	2.278	3.899	202.4	5.64	0.85

Table S2. The energy binary parameters (ξ_{ij}) for binary refrigerant mixtures of HFCs + HFC, HFC + HFO, and HFC + HCFO. The size binary parameter (η_{ij}) was fixed to unity in all cases, while ξ_{ij}= 1.000 denotes full predictions from the model without adjustment to available data. Parameters are transferred from our previous contribution.[3]

**Mixture**	ξ_{ij}	**Mixture**	ξ_{ij}	**Mixture**	ξ_{ij}
R32 + R23	1.000	R41 + R1234yf	0.980	R1123 + R1234yf	1.000
R32 + R161	1.050	R32 + R1123	0.985	R1123 + R1234ze(E)	1.000
R32 + R152a	0.975	R32 + R1234yf	0.970	R1243zf + R1234ze(E)	1.010
R32 + R134a	0.980	R32+ R1234ze(E)	1.000	R1234yf + R1234ze(E)	1.000
R32 + R125	0.985	R23 + R1234yf	0.970	R1234yf + R1233zd(E)	0.990
R32 + R236fa	1.000	R161 + R1234yf	1.010		
R32 + R227ea	1.000	R161 + R1234ze(E)	1.010		
R23 + R152a	1.000	R152a + R1243zf	1.000		
R23 + R134a	1.000	R152a + R1234yf	0.980		
R23 + R125	0.975	R152a + R1234ze(E)	1.010		
R23 + R227ea	0.960	R134a + R1243zf	0.980		
R161 + R134a	1.045	R134a + R1234yf	0.983		
R161 + R125	1.030	R134a + R1234ze(E)	1.000		
R161 + R227ea	1.030	R125 + R1234yf	1.000		
R152a +R134a	1.000	R125 + R1234ze(E)	1.000		
R152a + R125	1.000	R245fa + R1234ze(E)	1.000		
R152a + R245fa	1.000	R227ea + R1234yf	1.000		
R152a + R227ea	1.000	R227ea + R1234ze(E)	1.000		
R134a + R125	0.993				
R134a + R245fa	1.000				
R134a + R236fa	1.000				
R134a + R227ea	0.990				
R125 + R245fa	0.985				
R125 + R236fa	0.980				
R125 + R227ea	0.990				

References

(1) Albà, C. G.; Llovell, F.; Vega, L. F. Searching for Suitable Lubricants for Low Global Warming Potential Refrigerant R513A Using Molecular-Based Models: Solubility and Performance in Refrigeration Cycles. *Int. J. Refrig.* **2021**, *128*, 252–263. https://doi.org/10.1016/j.ijrefrig.2021.04.010.

(2) Albà, C. G.; Alkhatib, I. I. I.; Llovell, F.; Vega, L. F. Assessment of Low Global Warming Potential Refrigerants for Drop-In Replacement by Connecting Their Molecular Features to Their Performance. *ACS Sustain. Chem. Eng.* **2021**, *9* (50), 17034–17048. https://doi.org/10.1021/acssuschemeng.1c05985.

(3) Albà, C. G.; Alkhatib, I. I. I.; Llovell, F.; Vega, L. F. Hunting Sustainable Refrigerants Fulfilling Technical, Environmental, Safety and Economic Requirements. *Renew. Sustain. Energy Rev.* **2023**, *188*, 113806. https://doi.org/10.1016/j.rser.2023.113806.
